# Examining the Relationship between Heavy Alcohol Use and Assaults: With Adjustment for the Effects of Unmeasured Confounders

**DOI:** 10.1155/2015/596179

**Published:** 2015-08-25

**Authors:** Wenbin Liang, Tanya Chikritzhs

**Affiliations:** National Drug Research Institute, Curtin University, P.O. Box U1987, Perth, WA 6845, Australia

## Abstract

*Background*. Experimental studies suggest that alcohol can lead to aggression in laboratory settings; however, it is impossible to test the causal relationship between alcohol use and real-life violence among humans in randomized clinical trials. *Objectives*. (i) To examine the relationship between heavy alcohol use and assaults in a population based study; (ii) to demonstrate the proxy outcome method, as a means of controlling the effects of unknown/unmeasured confounders in observational studies. *Methods*. This study used data collected from three waves of the National Survey on Drug Use and Health (NSDUH). The effects of heavy alcohol use on assault were measured using multivariable logistic regressions in conjunction with the proxy outcome method. *Results*. Application of the proxy outcome method indicated that effect sizes of heavy alcohol use on the risk of assault were overestimated in the standard models. After adjusting for the effects of unknown/unmeasured confounders, the risk of assault remained 43% and 63% higher (*P* < 0.05) among participants who consumed 5+ drinks/day for 5–8 days/month and 9–30 days/month, respectively. *Conclusions*. Even after adjustment for unknown/unmeasured confounders the association between heavy alcohol use and risk of violence remained significant. These findings support the hypothesis that heavy alcohol use can cause violence.

## 1. Introduction

Substantial evidence from experimental studies suggests that alcohol can lead to aggression in laboratory settings [1–5] and validated laboratory methods to measure physical aggression such as the Taylor aggression paradigm [6] and the hot sauce procedure [7] have been well-developed. Nevertheless, it is difficult to generalize laboratory results to real-life occurrences of alcohol-related violence. It is impossible to test the causal relationship between alcohol use and physical violence that occurs among people in real life (such as assault) in randomized clinical trials due to ethical concerns. Data from observational studies have shown a positive association between alcohol use and violence in general populations. However, due to the nature of observational studies, it is difficult to conclude whether the observed association between alcohol use and violence is due to alcohol use or whether it is due to common cause factors [8–12]. For example, the well-designed longitudinal study by Fergusson and Horwood showed that cohort participants who had been diagnosed with alcohol abuse were more likely to be involved in violence and property crime. However, use of the data from their study alone was unable to determine whether alcohol caused the law-breaking behaviors or whether violence, property crime, and alcohol abuse were caused by common factors, for example, changes in mental health which may increase the likelihood of high-risk behaviors [13–16]. Therefore, further studies are required to test the hypothesis that heavy alcohol use causes violence.

Recent published works have demonstrated that unmeasured/unknown confounding effects could be estimated by measuring the association between the exposure variable (heavy alcohol use in this case) and a proxy outcome, on which the exposure has no or very limited effect [17–20]. The proxy outcome method is a general approach which provides estimates and adjustments for effects of unmeasured confounders. Many types of physical and mental health outcomes are affected by clusters of socioeconomic determinants and genetic and behavioural psychological factors [21–31]. Field specific knowledge and experience can be applied to determine the best proxy outcome for estimating effects of such unmeasured confounders. The papers by Tchetgen Tchetgen and Liang et al. provided detailed discussion on the methodological framework of the proxy outcome method [17, 19]. A recent study by Liang and Chikritzhs applied the proxy outcome method to an investigation of the effect of alcohol use on health status. They used the health status of drinkers' children as the proxy outcome to measure and control for unknown/unmeasured confounding effects that cluster within families such as socioeconomic determinants, environmental factors, lifestyle, and genetic susceptibility [18]. The findings from this study concurred with the results from a lately published Mendelian randomization analysis of pooled data from prospective studies that measured genetic variants [32]: both studies suggested no protective effect of moderate alcohol use on health.

**Figure 1:**
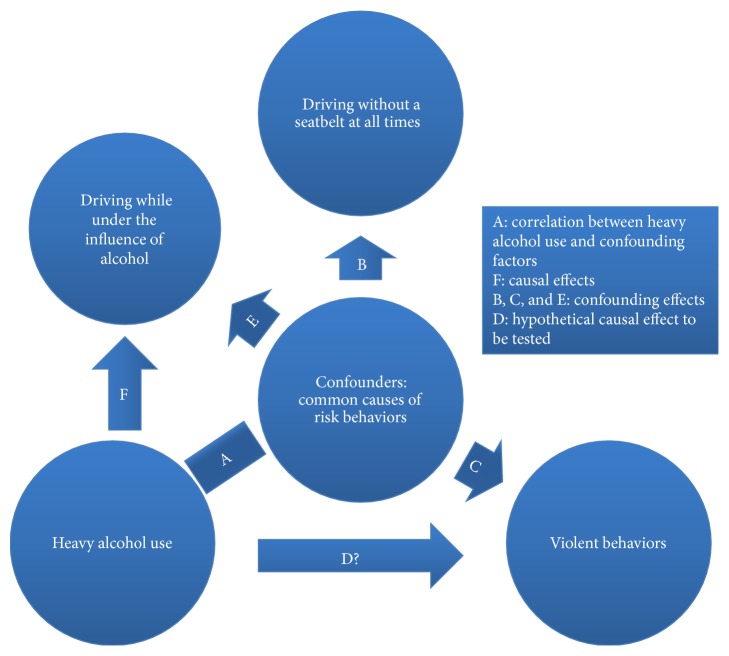
Illustration of an alternative approach to dealing with confounding effects.

In the current study, the proxy outcome method was adopted to control the effects of unknown confounders. The criteria for a proxy outcome are (i) the exposure of interest is not a cause for the proxy outcome and (ii) causes of the proxy outcome and the main outcome are subsets of a pool of correlated variables [17]. In order to avoid overadjustment, a positive outcome was used. The criteria for a positive outcome are (i) the exposure of interest is a cause for the positive outcome and (ii) causes of the positive outcome, the proxy outcome, and the main outcome are subsets of a pool of correlated variables. In relation to this study, driving without a seatbelt at all times met the criteria of a proxy outcome, and driving while under the influence of alcohol met the criteria of a positive outcome since (i) the three outcomes (violent behavior, driving without a seatbelt at all times, and driving while under the influence of alcohol) are all risk-taking illegal behaviors which share a set of similar social, environmental, and genetic risk factors [15, 33–35], as well as a similar direction and magnitude of response bias due to social desirability [36], and (ii) heavy alcohol use has minimum effect on driving without a seatbelt at all times while heavy alcohol use is an important cause of drink-driving (but not an essential cause, as some cases may be due to “moderate” level drinking) (also see Figure 1).

## 2. Materials and Methods

This study used data collected from three waves (2009, 2010, and 2011) of the National Survey on Drug Use and Health (NSDUH). NSDUH surveys are conducted to measure the prevalence and correlates of drug use in the United States. Details of the survey method have been described previously [37–39]. Briefly, NSDUH surveys are multistage national surveys with representative samples of noninstitutionalized population of the United States aged 12 years or older. In-home, computer-assisted interviews were used to collect the data. Since 2002, each participant who completed the survey was provided a $30 cash payment to improve the response rate. The response rates were 75.7%, 74.7%, and 74.4% for the 2009, 2010, and 2011 survey, respectively [37–39]. In addition to the questions on demographics and use of tobacco products, alcohol, and illicit drugs, participants were also asked questions about risk behaviors in the past 12 months and questions that assess mental and physical health conditions. This study included the samples from the 2009, 2010, and 2011 NSDUH surveys who were 18 years or older at the time of the survey, had consumed alcohol, and drove a car in the past 12 months.

Responses to the following questions were used to define violence, driving without seatbelt, and driving under the influence of alcohol: (1) violent behavior: “During the past 12 months, how many times have you attacked someone with the intent to seriously hurt them?”; (2) driving without seatbelt: “How often do you wear a seatbelt when you drive a car?”; and (3) driving under the influence of alcohol: “During the past 12 months, have you driven a vehicle while you were under the influence of alcohol?”. Answers to these questions were converted to three corresponding binary variables (yes/no) to represent the presence/absence of violent behavior (i.e., a yes if ever tried to seriously hurt someone in the past 12 months), driving without seatbelt at all times (yes if never wore a seatbelt when driving in the past 12 months), and driving under the influence of alcohol (yes if ever drove under the influence of alcohol in the past 12 months), respectively.

NSDUH survey referred a “drink” as a “can or bottle of beer, or a wine cooler, a shot of liquor, or a mixed drink with liquor in it” [37–39]. Number of days in which five or more drinks were consumed on the same occasion (occasion: “at the same time or within a couple of hours”) in the past 30 days prior to the interview was used as the measurement of heavy alcohol use. Number of days when consuming 5+ drinks over the past 30 days was converted into a six-category variable: 0 days, 1 day, 2 days, 3-4 days, 5–8 days, and 9–30 days. These cutoff points were chosen to ensure that while having as many categories as possible, the smallest sample size of a category was at least 5% of the total sample.

Multivariable logistic regressions were used to examine the relationships between the outcome variables (assault, driving without wearing seatbelt all of the time, and driving under the influence of alcohol) and heavy alcohol use while controlling for demographics (age, gender, race, marital status, and type of country of living); socioeconomic status (income and highest academic achievement); health (general health status). Whether ever had a lifetime major depressive episode, and if yes then whether there was an episode in the past 12 months which was assessed based on criteria in the Diagnostic and Statistical Manual of Mental Disorders 4th Edition (DSM-IV); and use of tobacco or any illicit drug in the past 12 months. The association between driving without wearing seatbelt and heavy alcohol use was used as a proxy estimate of unknown confounding effects toward the relationship between violence and heavy alcohol use as well as the relationship between driving under the influence of alcohol and heavy alcohol use. A final regression model was then performed to obtain the new estimates for violence and alcohol use that controlled for the confounding effects estimated by the proxy models. STATA 12 developed by StataCorp was used to perform the analysis. For further illustration, additional logistic regression models were fitted with only alcohol use as a predictor variable—treating all potential confounding factors as unknown (i.e., factors shown in Table 1, such as age, gender, and race) and leaving all confounding effects for the proxy outcome to account for.

There were 82,790 participants that met the selection criteria. Less than 1% of these participants did not provide necessary information on the dependent variables or some of the independent variables and therefore were excluded from the analysis (*n* = 82,272 remained in the analysis). The sampling weight supplied with the dataset was used in all analyses [37].

## 3. Results

Multivariable analysis showed that all of the three outcomes were significantly associated with heavy alcohol use after controlling for a number of known potential confounders (Table 1). The descriptive statistics are showed in Tables 4, 5, and 6. The effect size of heavy alcohol use was largest on driving under the influence of alcohol, which is known to be at least partly caused by heavy use (i.e., moderate levels of alcohol use may also be a cause of positive responses for driving under the influence of alcohol). The effect size of heavy alcohol use on violence was the second largest. The effect of heavy alcohol use on driving without a seatbelt at all times was smallest but remained significant. This provided a useful indicator of the magnitude of unknown/unmeasured confounding effects on the association between alcohol use and violent behavior.

The natural logarithms of the adjusted odds ratio for driving without a seatbelt by number of days with heavy alcohol use in the last 30 days were 0 (reference level) for 0 days, 0.12 for 1 day, 0.30 for 2 days, 0.42 for 3-4 days, 0.32 for 5–8 days, and 0.42 for 9 days or more, respectively. After offsetting the residual confounding effects, the effect of heavy alcohol use on violence became nonsignificant for 4 days' or less exposure in the last 30 days. Although reduced, the effects of 5–8 days' and 9+ days' heavy alcohol use on the risk of violence nevertheless remained significant. The estimates for the effects of heavy alcohol use on driving under the influence of alcohol were reduced but remained significant for all exposure categories (Table 2).

## 4. Discussion

This study examined the association between heavy alcohol use and risk of violence in uncontrolled settings (i.e., observational rather than experimental). The proxy outcome approach was employed to account for unknown/unmeasured confounding effects that may remain in estimates obtained using the standard approach. In both the standard and proxy outcome approaches it was observed that heavy alcohol use for 5 days or more in the past 30 days was significantly associated with increased risk of violence. This observation is consistent with findings from experimental studies which suggest that alcohol use may increase aggression in both humans and animals [1, 2, 40–42]. It has been hypothesized that physiological effects of alcohol on the central nervous system can impair cognitive functions that regulate emotion and behaviors [43, 44]. Individuals who have been suppressing angry feelings may express anger through acts of physical violence when self-control mechanisms are compromised due to alcohol use [11, 43–45].

The proxy outcome used in this study was driving without wearing a seatbelt at all times. Since not wearing a seatbelt at all times is not caused by alcohol, the apparent association is due to confounding. Similar confounding likely affected the apparent association between heavy alcohol use and violent behavior. Therefore, by controlling for confounding identified in the proxy outcome, we have more accurately described the magnitude of the true association between alcohol and violence. Thus, using a novel approach, this study has provided further evidence to support the notion that there is a causal relationship between alcohol use and violence.

We have described here an alternative approach to dealing with unknown/unmeasured confounding factors in observational studies that has application to the wider field of epidemiology. To further illustrate the application of this approach, we ran additional logistic regression models that were fitted with alcohol use as the only predictor (univariate model), thereby creating a hypothetical scenario where all potential confounding factors were unknown. We then applied the proxy outcome method as the only means of adjusting for potential confounding effects. As the estimates in Table 3 indicated, the confounding which dominated the crude results had been significantly removed using only the proxy outcome method and these estimates were, in fact, closely comparable to estimates derived using the standard approach that controlled for all known potential confounding factors (Table 1). Figure 1 was presented to further explain this approach. In order to measure “D” and “F” while adjusting for confounding, the effect size of “B” was used as approximation of the effect sizes of “C” and “E.” The certain causal effect of heavy alcohol use on driving under the influence of alcohol denoted as “F” was used as a positive control to detect whether any overadjustment had occurred.

The proxy outcome method is a general approach that enables analysts to control for the effects of unknown/unmeasured confounding factors. The advantage of this approach is that it will tend toward producing more conservative outcomes (i.e., effect sizes) than the standard approach which assumes that unknown/unmeasured confounding is minimal. However, under some circumstances, where the effect size of unmeasured confounding effects is severely overestimated, it may make a true causal effect (if it exists) undetectable. Therefore, a positive control outcome, such as the driving under the influence of alcohol variable used in this study, may be used to detect whether any overadjustment has occurred. In this case, after further adjustment, the effects of alcohol on the positive outcome remained significant at all levels of heavy alcohol use and therefore indicative that the true effect of alcohol exposure on violence is likely to remain detectable.

## 5. Conclusion

The association between heavy alcohol use and risk of violence remained significant after adjustment for the effects of known and unknown/unmeasured confounders. These findings support the hypothesis that heavy alcohol use is causal for violence. The novel proxy outcome method enabled the adjustment for the effects of unknown/unmeasured confounders and is a useful tool for improving the reliability of estimated effect sizes in observational research.

## Figures and Tables

**Table 1:** Associations between heavy alcohol use and three outcome variables (violent behaviour, driving without wearing a seatbelt all of the time, and driving under the influence of alcohol). Estimates from standard logistic regression.

	Violent behaviour			Driving without seatbelt			Driving while under the influence of alcohol		
	Odds ratio	95% CI		Odds ratio	95% CI^∧^		Odds ratio	95% CI^∧^	
Days of having 5+ drinks last month									
0	1.00			1.00			1.00		
1	1.22	0.96	1.56	1.13	0.91	1.41	2.50^*^	2.27	2.76
2	1.43^*^	1.06	1.93	1.35^*^	1.02	1.79	3.30^*^	2.96	3.68
3 to 4	1.45^*^	1.16	1.82	1.53^*^	1.20	1.95	4.60^*^	4.13	5.12
5 to 8	1.98^*^	1.58	2.47	1.38^*^	1.08	1.77	5.77^*^	5.15	6.47
9 to 30	2.49^*^	2.01	3.10	1.53^*^	1.21	1.93	6.88^*^	6.09	7.77

Used tobacco last year									
No	1.00			1.00			1.00		
Yes	1.45^*^	1.19	1.76	1.50^*^	1.25	1.80	1.12^*^	1.04	1.20

Used illicit drug last year									
No	1.00			1.00			1.00		
Yes	2.09^*^	1.80	2.44	1.10	0.94	1.28	2.66^*^	2.47	2.86

Age									
18	1.00			1.00			1.00		
19	0.86	0.69	1.08	0.87	0.66	1.15	1.03	0.88	1.19
20	0.70^*^	0.55	0.90	0.97	0.73	1.29	1.11	0.95	1.30
21	0.65^*^	0.52	0.83	1.02	0.77	1.35	1.29^*^	1.11	1.49
22–23	0.56^*^	0.45	0.70	1.30^*^	1.01	1.68	1.43^*^	1.25	1.63
24–25	0.49^*^	0.39	0.62	1.18	0.90	1.55	1.43^*^	1.24	1.64
26–29	0.37^*^	0.28	0.50	1.11	0.81	1.50	1.43^*^	1.23	1.67
30–34	0.31^*^	0.22	0.43	0.95	0.70	1.31	1.38^*^	1.18	1.62
35–49	0.17^*^	0.12	0.24	0.68^*^	0.50	0.93	1.22^*^	1.05	1.42
50–64	0.10^*^	0.06	0.17	0.49^*^	0.33	0.72	1.10	0.93	1.30
65+	0.14^*^	0.05	0.38	0.48^*^	0.28	0.82	0.66^*^	0.52	0.84

Gender									
Male	1.00			1.00			1.00		
Female	0.68^*^	0.59	0.79	0.44^*^	0.37	0.52	0.69^*^	0.64	0.73

Race									
White	1.00			1.00			1.00		
Black	2.05^*^	1.69	2.47	0.60^*^	0.47	0.76	0.81^*^	0.73	0.91
Native American	1.91^*^	1.23	2.95	0.75	0.40	1.41	1.32	0.91	1.92
Native Pacific Islander	3.88	0.92	16.41	0.50	0.14	1.85	0.73	0.34	1.53
Asian	1.00	0.61	1.64	0.73	0.35	1.49	0.48^*^	0.40	0.58
Mixed race	1.88^*^	1.27	2.78	0.87	0.56	1.36	0.76^*^	0.62	0.93
Hispanic	1.29^*^	1.03	1.60	0.48^*^	0.38	0.61	0.78^*^	0.70	0.87

Education									
Less than high school	1.00			1.00			1.00		
High school graduate	0.83	0.68	1.02	1.02	0.83	1.25	1.32^*^	1.18	1.49
Some college	0.55^*^	0.44	0.69	0.59^*^	0.47	0.74	1.68^*^	1.49	1.90
College graduate	0.36^*^	0.26	0.50	0.28^*^	0.21	0.39	2.16^*^	1.90	2.45

Family income									
<$20,000	1.00			1.00			1.00		
$20,000–$49,999	1.07	0.91	1.26	0.93	0.76	1.14	1.28^*^	1.16	1.40
$50,000–$74,999	0.74^*^	0.60	0.92	0.82	0.64	1.04	1.58^*^	1.42	1.77
$75,000+	0.78^*^	0.63	0.97	0.77^*^	0.61	0.98	1.70^*^	1.53	1.88

Marital status									
Married	1.00			1.00			1.00		
Widowed	0.98	0.35	2.74	1.64	0.89	3.06	0.86	0.65	1.13
Divorced or separated	1.42^*^	1.05	1.94	1.50^*^	1.18	1.90	1.30^*^	1.17	1.45
Never been married	1.38^*^	1.07	1.77	1.26^*^	1.02	1.55	1.24^*^	1.14	1.36

Health status									
Excellent	1.00			1.00			1.00		
Very good	0.95	0.79	1.15	0.99	0.82	1.20	1.11	1.02	1.20
Good	1.16	0.94	1.42	1.29^*^	1.06	1.57	0.94	0.86	1.03
Fair	1.16	0.88	1.54	1.60^*^	1.21	2.12	0.78^*^	0.67	0.90
Poor	3.32^*^	1.53	7.19	3.48^*^	1.91	6.35	0.39^*^	0.29	0.55

Major depressive episode									
Never	1.00			1.00			1.00		
Ever, but not in the last 12 months	1.51^*^	1.13	2.01	1.11	0.78	1.58	1.58^*^	1.41	1.77
In the last 12 months	3.02^*^	2.52	3.62	1.28	0.98	1.68	1.89^*^	1.70	2.10

County metro status									
Large metro	1.00			1.00			1.00		
Small metro	1.05	0.89	1.23	0.95	0.80	1.13	1.12^*^	1.04	1.20
Nonmetro	1.22	0.99	1.50	1.32^*^	1.08	1.60	1.02	0.94	1.12

Year of survey									
2009	1.00			1.00			1.00		
2010	0.92	0.77	1.09	1.00	0.85	1.19	0.95	0.89	1.03
2011	0.84	0.70	1.00	1.05	0.88	1.26	0.99	0.91	1.07

^∧^95% confidence interval.

^∗^ *P* < 0.05.

**Table 2:** Associations between heavy alcohol use and two outcome variables (assault and driving under the influence of alcohol). Estimates from standard logistic regression plus additional control for the effects of unmeasured confounders.

	Violent behaviour			Driving while under the influence of alcohol		
	Odds ratio	95% CI^∧^		Odds ratio	95% CI^∧^	
Days of having 5+ drinks last month						
0	1.00			1.00		
1	1.08	0.85	1.38	2.22^*^	2.01	2.44
2	1.06	0.78	1.43	2.44^*^	2.19	2.72
3 to 4	0.95	0.76	1.19	3.01^*^	2.70	3.35
5 to 8	1.43^*^	1.15	1.79	4.18^*^	3.73	4.69
9 to 30	1.63^*^	1.31	2.03	4.50^*^	3.99	5.08

Model controlled for the same potential confounding factors listed in Table 1 plus additional control for the effects of unmeasured confounders derived from proxy models.

^∧^95% confidence interval.

^∗^ *P* < 0.05.

**Table 3:** Comparison of estimates derived from standard univariate models and estimates derived from univariate models with proxy outcome method alone to account for all confounding effects.

	Violent behaviour			Driving without seatbelt			Driving while under the influence of alcohol		
	Odds ratio	95% CI		Odds ratio	95% CI		Odds ratio	95% CI^∧^	
Estimates from univariate models									
Days of having 5+ drinks last month									
0	1.00			1.00			1.00		
1	1.99^*^	1.57	2.52	1.57^*^	1.25	1.96	2.94^*^	2.68	3.21
2	2.73^*^	2.09	3.56	2.14^*^	1.64	2.79	4.02^*^	3.64	4.44
3 to 4	3.17^*^	2.57	3.91	2.68^*^	2.14	3.35	6.01^*^	5.46	6.62
5 to 8	4.62^*^	3.75	5.70	2.64^*^	2.08	3.36	8.31^*^	7.51	9.19
9 to 30	6.53^*^	5.38	7.94	3.55^*^	2.87	4.39	10.20^*^	9.17	11.34

Estimates from univariate models with proxy outcome method									
Days of having 5+ drinks last month									
0	1.00						1.00		
1	1.27	1.00	1.61				1.88^*^	1.71	2.05
2	1.27	0.98	1.66	Estimates not applicable for proxy outcome			1.88^*^	1.70	2.07
3 to 4	1.18	0.96	1.46	(will always be equal to 1)			2.25^*^	2.04	2.47
5 to 8	1.75^*^	1.42	2.15				3.14^*^	2.84	3.47
9 to 30	1.84^*^	1.52	2.24				2.87^*^	2.58	3.20

^∧^95% confidence interval.

^∗^ *P* < 0.05.

**Table 4:** Descriptive frequency statistics for violent behaviours, alcohol use, and controlled potential confounders.

	Violent behaviour		
	Yes	No	Total
Days of having 5+ drinks last month			
0	45,069	842	45,911
1	10,356	293	10,649
2	7,102	306	7,408
3 to 4	6,800	334	7,134
5 to 8	5,672	341	6,013
9 to 30	4,693	464	5,157

Used tobacco last year			
No	40,005	571	40,576
Yes	39,687	2,009	41,696

Used illicit drug last year			
No	56,921	946	57,867
Yes	22,771	1,634	24,405

Age			
18	4,144	395	4,539
19	4,374	322	4,696
20	4,689	268	4,957
21	5,419	311	5,730
22–23	10,500	460	10,960
24–25	10,367	354	10,721
26–29	6,046	152	6,198
30–34	6,601	127	6,728
35–49	17,165	155	17,320
50–64	7,234	27	7,261
65+	3,153	9	3,162

Gender			
Male	38,700	1,608	40,308
Female	40,992	972	41,964

Race			
White	54,968	1,392	56,360
Black	8,197	508	8,705
Native American	1,020	78	1,098
Native Pacific Islander	313	18	331
Asian	2,525	43	2,568
Mixed race	2,253	129	2,382
Hispanic	10,416	412	10,828

Education			
Less than high school	9,125	663	9,788
High school graduate	24,301	1,096	25,397
Some college	25,729	659	26,388
College graduate	20,537	162	20,699

Family income			
<$20,000	17,994	881	18,875
$20,000–$49,999	26,213	978	27,191
$50,000–$74,999	13,366	321	13,687
$75,000+	22,119	400	22,519

Marital status			
Married	28,638	289	28,927
Widowed	1,266	11	1,277
Divorced or separated	7,376	187	7,563
Never been married	42,412	2,093	44,505

Health status			
Excellent	21,488	557	22,045
Very good	32,969	980	33,949
Good	19,481	762	20,243
Fair	4,971	243	5,214
Poor	783	38	821

Major depressive episode			
Never	67,989	1,908	69,897
Ever, but not in the last 12 months	5,450	176	5,626
In the last 12 months	6,253	496	6,749

County metro status			
Large metro	34,746	1,052	35,798
Small metro	28,703	968	29,671
Nonmetro	16,243	560	16,803

Year of survey			
2009	26,084	929	27,013
2010	27,080	872	27,952
2011	26,528	779	27,307

**Table 5:** Descriptive frequency statistics for driving without seatbelt, alcohol use and controlled potential confounders.

	Driving without seatbelt		
	No	Yes	Total
Days had 5+ drinks last month			
0	44,888	1,023	45,911
1	10,308	341	10,649
2	7,092	316	7,408
3 to 4	6,786	348	7,134
5 to 8	5,691	322	6,013
9 to 30	4,792	365	5,157

Used tobacco last year			
No	39,911	665	40,576
Yes	39,646	2,050	41,696

Used illicit drug last year			
No	56,381	1,486	57,867
Yes	23,176	1,229	24,405

Age			
18	4,314	225	4,539
19	4,474	222	4,696
20	4,741	216	4,957
21	5,481	249	5,730
22–23	10,490	470	10,960
24–25	10,297	424	10,721
26–29	5,987	211	6,198
30–34	6,561	167	6,728
35–49	16,950	370	17,320
50–64	7,143	118	7,261
65+	3,119	43	3,162

Gender			
Male	38,425	1,883	40,308
Female	41,132	832	41,964

Race			
White	54,303	2,057	56,360
Black	8,474	231	8,705
Native American	1,056	42	1,098
Native Pacific Islander	325	6	331
Asian	2,539	29	2,568
Mixed race	2,294	88	2,382
Hispanic	10,566	262	10,828

Education			
Less than high school	9,169	619	9,788
High school graduate	24,181	1,216	25,397
Some college	25,696	692	26,388
College graduate	20,511	188	20,699

Family income			
<$20,000	18,159	716	18,875
$20,000–$49,999	26,110	1,081	27,191
$50,000–$74,999	13,286	401	13,687
$75,000+	22,002	517	22,519

Marital status			
Married	28,363	564	28,927
Widowed	1,248	29	1,277
Divorced or Separated	7,273	290	7,563
Never been married	42,673	1,832	44,505

Health status			
Excellent	21,479	566	22,045
Very good	33,005	944	33,949
Good	19,356	887	20,243
Fair	4,958	256	5,214
Poor	759	62	821

Major depressive episode			
Never	67,589	2,308	69,897
Ever, but not in the last 12 months	5,483	143	5,626
In the last 12 months	6,485	264	6,749

County metro status			
Large metro	34,873	925	35,798
Small metro	28,748	923	29,671
Nonmetro	15,936	867	16,803

Year of survey			
2009	26,083	930	27,013
2010	27,049	903	27,952
2011	26,425	882	27,307

**Table 6:** Descriptive frequency statistics for driving while under the influence of alcohol, alcohol use and controlled potential confounders.

	Driving while under the influence of alcohol		
	No	Yes	Total
Days had 5+ drinks last month			
0	40,715	5,196	45,911
1	8,010	2,639	10,649
2	5,026	2,382	7,408
3 to 4	4,271	2,863	7,134
5 to 8	3,079	2,934	6,013
9 to 30	2,188	2,969	5,157

Used tobacco last year			
No	34,317	6,259	40,576
Yes	28,972	12,724	41,696

Used illicit drug last year			
No	48,963	8,904	57,867
Yes	14,326	10,079	24,405

Age			
18	3,544	995	4,539
19	3,518	1,178	4,696
20	3,698	1,259	4,957
21	4,092	1,638	5,730
22–23	7,717	3,243	10,960
24–25	7,664	3,057	10,721
26–29	4,587	1,611	6,198
30–34	5,199	1,529	6,728
35–49	14,100	3,220	17,320
50–64	6,212	1,049	7,261
65+	2,958	204	3,162

Gender			
Male	29,239	11,069	40,308
Female	34,050	7,914	41,964

Race			
White	42,468	13,892	56,360
Black	7,149	1,556	8,705
Native American	814	284	1,098
Native Pacific Islander	255	76	331
Asian	2,131	437	2,568
Mixed race	1,811	571	2,382
Hispanic	8,661	2,167	10,828

Education			
Less than high school	8,019	1,769	9,788
High school graduate	20,081	5,316	25,397
Some college	19,756	6,632	26,388
College graduate	15,433	5,266	20,699

Family income			
<$20,000	14,557	4,318	18,875
$20,000–$49,999	21,107	6,084	27,191
$50,000–$74,999	10,530	3,157	13,687
$75,000+	17,095	5,424	22,519

Marital status			
Married	24,193	4,734	28,927
Widowed	1,157	120	1,277
Divorced or separated	5,994	1,569	7,563
Never been married	31,945	12,560	44,505

Health status			
Excellent	17,083	4,962	22,045
Very good	25,544	8,405	33,949
Good	15,670	4,573	20,243
Fair	4,277	937	5,214
Poor	715	106	821

Major depressive episode			
Never	54,700	15,197	69,897
Ever, but not in the last 12 months	4,029	1,597	5,626
In the last 12 months	4,560	2,189	6,749

County metro status			
Large metro	27,777	8,021	35,798
Small metro	22,535	7,136	29,671
Nonmetro	12,977	3,826	16,803

Year of survey			
2009	20,449	6,564	27,013
2010	21,565	6,387	27,952
2011	21,275	6,032	27,307
